# Regional variation in breast cancer treatment in the Netherlands and the role of external peer review: a cohort study comprising 63,516 women

**DOI:** 10.1186/1471-2407-14-596

**Published:** 2014-08-16

**Authors:** Melvin J Kilsdonk, Boukje AC van Dijk, Renee Otter, Wim H van Harten, Sabine Siesling

**Affiliations:** Department of Research, Comprehensive Cancer Centre the Netherlands, Postbus 19079, 3501 DB Utrecht, The Netherlands; School for Management and Governance, Department Health Technology and Services Research, University of Twente, Enschede, The Netherlands; Department of Epidemiology, University of Groningen, University Medical Centre Groningen, Groningen, The Netherlands; The Netherlands Cancer Institute, Amsterdam, The Netherlands

**Keywords:** Breast neoplasms, Cohort studies, Healthcare quality assessment, Quality improvement, Peer review

## Abstract

**Background:**

Treatment variation is an important issue in health care provision. An external peer review programme for multidisciplinary cancer care was introduced in 1994 in the Netherlands to improve the multidisciplinary organisation of cancer care in hospitals.

So far the clinical impact of external quality assessment programmes such as external peer review and accreditation remains unclear. Our objective was to examine the degree of variation in treatment patterns and the possible effect of external peer review for multidisciplinary cancer care for breast cancer patients.

**Methods:**

Patients with breast cancer were included from 23 hospitals from two ‘intervention regions’ with the longest experience with the programme and 7 hospitals that never participated (control group). Data on tumour and treatment characteristics were retrieved from the Netherlands Cancer Registry. Treatment modalities investigated were: the completeness of breast conserving therapy, introduction of the sentinel node biopsy, radiotherapy after breast conserving surgery for ductal carcinoma in situ (DCIS), adjuvant radiotherapy for locally advanced breast cancer (T3/M0 or any T,N2-3/M0), adjuvant chemotherapy for early stage breast cancer (T1-2/N+/M0) and neo-adjuvant chemotherapy for T4/M0 breast cancer. Hospitals from the two intervention regions were dichotomised based on their implementation proportion (IP) of recommendations from the final reports of each peer review (high IP vs. low IP). This was regarded as a measure of how well a hospital participated in the programme.

**Results:**

63,516 female breast cancer patients were included (1990-2010). Variation in treatment patterns was observed between the intervention regions and control group. Multidisciplinary treatment patterns were not consistently better for patients from hospitals with a high IP.

**Conclusions:**

There is no relationship between the external peer review programme for multidisciplinary cancer care and multidisciplinary treatment patterns for breast cancer patients. Regional factors seem to exert a stronger effect on treatment patterns than hospital participation in external peer review.

**Electronic supplementary material:**

The online version of this article (doi:10.1186/1471-2407-14-596) contains supplementary material, which is available to authorized users.

## Background

Breast cancer is the commonest cancer in women in the Netherlands and its burden increased during the last decades due to a steady rise in incidence [1]. Survival rates have improved because of better imaging and detection techniques, screening programmes and the introduction of new therapies [2, 3]. Breast cancer treatment is marked by a multidisciplinary approach and specialisation of the involved medical and nursing specialists. A recent study in 13,722 women showed that improving multidisciplinary care was associated with improved survival and reduced variation in survival among hospitals [4]. Specialisation of physicians is an important component of multidisciplinary care and is associated with better outcomes for various cancers [5]. A study in the UK revealed an 11-17% reduction in risk of death in women treated for breast cancer as a result of specialisation of surgeons [6]. Similar results were seen in other types of cancer and during the 90’s multidisciplinary care became the standard of cancer care. It is known that treatment variation exists between and within countries and it is unknown whether and how these differences interact with improvement efforts. This poses serious challenges in efforts to evaluate quality improvement programmes.

Several quality improvement methods are used to improve the multidisciplinary organisation of care and reduction of variation. In the Netherlands an external peer review programme was introduced in 1994. Designed and executed by medical and nursing cancer specialists, it was introduced in the Northern Netherlands and gradually spread over the entire country. The programme focuses on the organisational conditions to provide optimal cancer care. Participation is voluntary and hospitals are advised to participate in cycles of 4–5 years. After a self-assessment, on-site observation and interviews, the organisation of cancer care in a hospital is evaluated and recommendations for improvement are given. Major topics of recommendations were the organisation of weekly multidisciplinary patient care meetings, shared decision making between specialists, oncological specialisation of medical specialists, dedication of oncology committees (with representatives of all medical specialisms) to policy making, referral policies for rare tumours and highly complicated interventions, introduction of integrated care pathways and working to evidence based guidelines. More information on the programme can be found in Additional file 1.

In general, the clinical impact of external peer review remains under-investigated. A study evaluating a peer review programme for chronic obstructive pulmonary disease in the United Kingdom found an association with improved quality of care, service delivery and changes that promote quality improvement after three years [7]. The evaluation after one year revealed no differences showing that changes in healthcare can take a prolonged period to occur [8]. Accreditation is the most frequently studied form of external quality assessment. Literature reviews on the effects of accreditation on the quality of care could not provide strong evidence due to limitations of the studies [9, 10]. The programmes demand high financial and labour investments and therefore there is a need for more research on the clinical impact of these programmes [11, 12].

The purpose of our study was to investigate the multidisciplinary treatment patterns of breast cancer patients and the effect of the external peer review programme for multidisciplinary cancer care in general hospitals. In a previous study we found some positive effects on colorectal cancer treatment, but the results needed to be interpreted cautiously due to the ambiguity of the outcomes and possible confounding factors [13]. In the current study we examined whether our previous results are also evident in breast cancer treatment. More importantly, by analysing different regions separately we hope to gain more insights in possible regional confounders. We hypothesised that the willingness of a hospital to have external peer review and to follow the recommendations from it, is correlated to the hospital giving higher quality of breast cancer treatment measured by the introduction of new multidisciplinary therapies.

## Methods

### Design and patients

Only female patients diagnosed with primary epithelial breast cancer (ICD-O 10, International Classification of Diseases, codes: C50.0 to 50.9) between 1 January 1990 and 31 December 2010 were selected from the Netherlands Cancer Registry (NCR). This is a population based independent cancer registry containing clinical administrative data of every newly diagnosed cancer patient in the Netherlands. Data is collected directly from the hospitals’ patient files by specially trained registration clerks. Topography and morphology is coded according to the International Classification of Diseases for Oncology (ICD-O) and staging according to the TNM-classification. Follow-up of vital status is achieved by linkage of the registry to municipal records. The quality of the data is high [14] and completeness is estimated to be at least 95% [15].

Patients were included from hospitals in the Northern Netherlands and the Rotterdam region. In these regions the external peer review programme was introduced first (intervention regions). Patients from hospitals from other regions that never participated before 2009 were included in the control group. We excluded patients that were diagnosed with neuroendocrine tumours, synchronous tumours, diagnosed at autopsy and that had any type of previous malignancy.

### Hospital categories

Hospitals from the intervention group were categorised by the implementation proportion (IP) of recommendations that were given in the final reports of each peer review. We dichotomised the intervention region hospitals by their IP (high IP vs. low IP, no threshold was used). We regarded the IP of the recommendations as a proxy of how well a hospital participated in the programme. Rating the implementation was performed by studying final reports from subsequent reviews, follow-up correspondence, hospital documents and interviews with stakeholders when necessary. Implementation of a recommendation was ranked on a scale from 0 to 4 (Table 1). The IP per hospital was expressed as a percentage of the total possible score. When implementation of a recommendation could not be determined (lost to follow-up), this recommendation was subtracted from the total possible score. The average IP of all peer reviews per hospital was used because it is not known what the time period is in which changes based on organisational change can occur and quality improvement is a continuous process. Ranking the implementation of recommendations was performed by the principal investigator. If e.g. the report from the next peer-review states that a recommendation was not implemented at all this was ranked as zero. Full implementation was ranked as 4, examples of recommendations and their ranking can be seen in Table 1. Due to the objective nature of the evidence the ranking was not considered to be arbitrarily and we did not use an inter-rater approach.

**Table 1:** **Criteria and (real) examples of the ranking of implementation of the recommendations on a scale from 0-4**

Implementation score	Criteria	Recommendation	Follow up report
0	Not implemented at all		
1	Hospital only started working on implementing	The oncology committee should make oncological policy plans	An oncological policy plan is in preparation
2	A recommendation consists of two parts and one is implemented	An oncology committee needs to be formed consisting of physicians and a nursing staff representative	There is an oncology committee consisting of physicians but no nursing staff representative
3	Recommendation is implemented but not yet in the entire organisation	There should be oncological specialisation, especially amongst the surgeons, urologists and gynacologists	Oncological specialisation was realised in surgery, gynaecology, internal and pulmonary medicine but not in urology.
4	Complete implementation	The hospital should have a fulltime pulmonary physician if lung surgery is performed for an optimal pre-, peri- and post-operative care	The hospital appointed a full-time pulmonary physician

From the hospitals in the two intervention regions we used data from two or three cycles of participation:

- Northern Netherlands: three cycles, 1994–2009.
- Rotterdam region: two cycles, 1996–2006. A third cycle was completed between 2009 and 2011 but follow-up time was too short to monitor the IP.

All hospitals in these regions voluntarily participated in the peer review programme. The university medical centres and hospitals that merged during our study period were excluded, because it was impossible to follow-up the recommendations. Hospitals were asked to participate in the study by giving permission to use their data from the NCR and final reports.

### Analyses

We analysed the Northern Netherlands and Rotterdam region separately to gain more insights in possible regional confounders besides the external peer review programme. Patients were grouped according to the hospital in which the diagnosis was made. They may have been referred for treatment but this was regarded to be good clinical practice (and referral policy is a theme of the programme). Multivariate logistic analysis was used to analyse treatment variation and the influence of hospital category (based on IP), gender, age at diagnosis, year of diagnosis, average hospital volume of diagnoses and presence of an in-hospital radiotherapy department. We studied several multidisciplinary treatment modalities. First of all, we studied *the completeness of breast conserving therapy (BCT)*. From its introduction onwards, breast conserving therapy is a multidisciplinary procedure and one of the earliest examples of multidisciplinary cancer treatment. Breast conserving surgery (BCS) was initially complemented with axillary lymph node dissection (ALND) and radiotherapy. Omission of lymph node dissection is allowed after a negative sentinel node biopsy (SNB). In our analyses, BCT was considered complete if radiotherapy had been given and ALND was performed or when radiotherapy is given, SNB was performed and ALND was omitted. We separately analysed the *introduction of the sentinel node biopsy*. Other indicators for treatment variation were taken from the indicator list defined by the NABON (National Breast Cancer Network Netherlands) in 2009. This list is part of a national audit on the quality of breast cancer diagnostics and treatment (NBCA) that started in 2011 [16]. These indicators are: *radiotherapy after BCS for ductal carcinoma in situ (DCIS)*, *adjuvant radiotherapy for locally advanced breast cancer* (T3/M0 or any T,N2-3/M0), *adjuvant chemotherapy for early stage breast cancer* (T1-2/N+/M0) and *neo-adjuvant chemotherapy for T4/M0 breast cancer*. Although the NBCA was established in 2011, data on the selected indicators were available since 1990. We could therefore look in retrospect at the period from 1990 onwards to evaluate how hospitals performed on these quality indicators that we now regard to be the standard of care for breast cancer patients.

For the analyses of completeness of breast conserving therapy and adjuvant chemotherapy for early stage breast cancer pathological stage was used and substituted with clinical stage if pathological stage was unknown. For the rest of the analyses clinical stage was used substituted by pathological stage if unknown. STATA version 12.0 was used for all analyses. Written syntaxes guarantee reproducibility of the results. P values were considered significant if smaller than 0.05.

## Results

### Hospitals and recommendations

Twenty-six hospitals from the Northern Netherlands and Rotterdam region were asked to give permission to use the data from their peer reviews and the Netherlands Cancer Registry. Twenty-three gave permission: 13 hospitals from the Northern Netherlands and 10 from the Rotterdam region. Seven out of twelve hospitals without experience with the programme agreed to be included in the control group. In total, our study includes patient data from 30 hospitals, approximately one-third of all hospitals in the Netherlands. In the three cycles of peer review in the Northern Netherlands and two cycles in the Rotterdam region 727 recommendations were given, averaging 12 recommendations per peer review per hospital. The intervention hospitals in both regions were dichotomised based on the IP of the recommendations. The Northern Netherlands region was divided in 6 hospitals with a high IP (average IP 63.2%) and 7 hospitals with a low IP (average IP 48.9%). The Rotterdam region was dichotomised in 5 hospitals with a high IP (average IP 63.2%) and 5 with a low IP (average 41.4%).

### Patients

Our total cohort consists of 63,516 women. Table 2 shows the characteristics of the population grouped by their hospital category. There were no large differences in mean age at diagnosis and the number of patients per period of diagnosis between patients diagnosed in the different hospital categories. The average annual case volume differs between the regions, as in the Rotterdam region no hospitals with less than 50 patients diagnosed annually existed in the period under study. For only two hospital categories hospitals with more than 100 diagnosis per year existed (Northern Netherlands high IP and control group, Table 2).

**Table 2:** **Characteristics of the study cohort**

Variable	North high IP ***N(%)***	North low IP ***N(%)***	Rotterdam high IP ***N(%)***	Rotterdam low IP ***N(%)***	Controls ***N(%)***
	***6 hospitals***	***7 hospitals***	***5 hospitals***	***5 hospitals***	***7 hospitals***
*Mean age at diagnosis*	61.16	61.48	61.33	61.40	59.80
	SD 14.16	SD 14.20	SD 14.34	SD 14.39	SD 13.67
*Period of diagnosis*					
1990-1995	3260 (23.21)	2095 (19.42)	2310 (23.16)	2249 (23.28)	4454 (23.38)
1996-2001	4079 (29.05)	3085 (28.60)	2717 (27.24)	2635 (27.28)	5131 (26.93)
2002-2007	4426 (31.52)	3558 (32.98)	3082 (30.90)	3044 (31.51)	5995 (31.47)
2008-2010	2278 (16.22)	2050 (19.00)	1866 (18.71)	1732 (17.93)	3470 (18.22)
Stage					
IS	1097 (7.81)	776 (7.19)	794 (7.96)	775 (8.02)	1577 (8.28)
1	4758 (33.88)	3660 (33.93)	3323 (33.31)	3249 (33.63)	6595 (34.62)
2	5881 (41.88)	4575 (42.41)	4243 (42.54)	4097 (42.41)	7898 (41.46)
3	1480 (10.54)	1123 (10.41)	1018 (10.21)	959 (9.93)	1835 (9.63)
4	718 (5.11)	547 (5.07)	513 (5.14)	487 (5.04)	877 (4.60)
X	109 (0.78)	107 (0.99)	84 (0.84)	93 (0.96)	268 (1.41)
Average annual volume of hospital of diagnosis					
<50	924 (6.58)	647 (6.00)	0 (0.00)	0 (0.00)	953 (5.00)
50-100	4226 (30.09)	10141 (94.00)	9975 (100)	9660 (100)	16.579 (87.03)
100 or more	8893 (63.33)	0 (0.00)	0 (0.00)	0 (0.00)	1518 (7.97)

Characteristics of breast cancer patients according to the hospital category, 1990–2010, data are no (%), *N* = 63,516. IP = Implementation Proportion of recommendations given in the programme.

### Completeness of breast conserving therapy

Incomplete breast conserving therapy, omitting radiotherapy and/or ALND after breast conserving surgery rarely occurred (Table 3). Although the absolute risk is low, the odd’s ratio’s show that the odd’s of receiving complete BCT were higher in both hospital categories in the Northern Netherlands.

**Table 3:** **Chances of receiving multidisciplinary treatment**

Treatment	Hospital category	% of patients treated according to guidelines				OR	95% CI
		‘90-‘95	‘96-‘01	‘02-‘07	‘08-‘10		
Complete breast conserving treatment. *N* = 22453	Controls	95.6	92.3	93.4	95.8	1.00	Reference
*Inclusion criteria: Stage I-IIIA*	North High IP	97.8	97.1	97.8	98.3	2.68*	2.08-3.45
*Exclusion criterium: Breast amputation after BCS*	North Low IP	95.4	96.3	96.7	97.1	1.77*	1.43-2.17
	Rotterdam High IP	93.0	88.9	91.9	96.7	0.77*	0.64-0.92
	Rotterdam Low IP	94.6	89.6	91.7	95.0	0.72*	0.60-0.85
Introduction of the SNB. *N* = 25612	Controls	0	33.9	93.6	98.3	1.00	Reference
*Inclusion criteria: cT1-2, cN0, cM0, BCS*	North high IP	0	16.2	93.9	98.8	0.68*	0.55-0.84
	North low IP	0	20.9	93.2	98.2	0.59*	0.50-0.70
	Rotterdam high IP	0	19.1	89.0	98.8	0.46*	0.38-0.55
	Rotterdam low IP	0	14.8	92.1	96.9	0.48*	0.40-0.57
Radiotherapy after BCS for DCIS. *N* = 2414	Controls	16.1	31.7	76.8	85.2	1.00	Reference
*Inclusion criteria: DCIS, BCS*	North high IP	50.0	49.0	74.8	84.9	1.24	0.88-1.74
	North low IP	57.1	43.7	72.5	81.7	1.13	0.84-1.52
	Rotterdam high IP	33.3	48.9	72.0	79.8	1.08	0.79-1.49
	Rotterdam low IP	27.6	50.4	74.1	83.3	1.27	0.93-1.73
Adjuvant radiotherapy locally advanced breast cancer *N* = 1511	Controls	64.6	67.3	64.6	69.1	1.00	Reference
*Inclusion criteria: cT3,anyN,M0 and any T,N2-3,M0 + amputation*	North high IP	53.5	54.4	53.5	68.3	0.75	0.51-1.10
	North low IP	46.2	62.5	46.2	53.2	0.56*	0.39-0.80
	Rotterdam high IP	39.2	34.2	39.2	61.5	0.40*	0.29-0.55
	Rotterdam low IP	29.7	35.9	29.7	68.6	0.36*	0.25-0.52
Adjuvant chemotherapy early stage breast cancer. *N* = 9511	Controls	51.3	73.1	85.1	91.9	1.00	Reference
*Inclusion criteria: pT1-2 M0/X, surgery age < 60*	North high IP	62.3	69.7	90.4	93.0	1.24	1.00-1.54
	North low IP	52.8	70.6	91.4	91.3	1.29*	1.07-1.54
	Rotterdam high IP	60.2	72.3	88.0	95.6	1.50*	1.26-1.81
	Rotterdam low IP	55.3	67.2	90.1	96.0	1.22*	1.01-1.46
Neo-adjuvant chemotherapy T4/M0 breast cancer. *N* = 1484	Controls	5.1	27.4	34.5	61.9	1.00	Reference
*Inclusion criteria: cT4NxM0, surgery*	North high IP	11.2	25.0	56.0	65.3	1.24	0.73-2.09
	North low IP	4.8	22.8	57.8	44.0	1.57*	1.00-2.47
	Rotterdam high IP	6.7	28.0	55.0	51.9	2.67*	1.74-4.07
	Rotterdam low IP	5.3	29.2	54.8	61.5	2.02*	1.32-3.08

Odd’s ratio’s for receiving multidisciplinary therapy per hospital category. Adjusted for age, year of incidence, annual volume of diagnoses per hospital, stage (if necessary). 1990–2010 *P < 0.05. IP = implementation proportion of recommendations given in the programme.

### Introduction of the SNB

Since 2003 guidelines recommend the SNB to be performed in T1-2/N0 breast cancer. Unfortunately, the SNB was not registered consistently in the NCR in some regions of the country. When an ALND was performed after SNB then only the ALND has been registered in these regions. This might give an underestimation of the group that had a BCT with SNB followed by ALND. In our study, this only concerns the control group. We excluded all patients from the control group that were diagnosed in hospitals with this deviating registration policy (*N* = 1950). The control group remained the largest group. Patients in the control region were more likely to receive a sentinel node biopsy compared to both intervention regions. The differences were most prominent between 1996–2001 (Table 3).

### Radiotherapy after BCS for DCIS

The total numbers of patients are low in the early periods. After the introduction of a nationwide screening programme the incidence of DCIS has gradually risen because of the increasing quality of diagnostics. In the latest time period the percentage of radiotherapy was over 79% in all hospital categories. No significant differences were seen between the odd’s for receiving radiotherapy in the different hospital categories (Table 3).

### Adjuvant radiotherapy for locally advanced breast cancer

Official guideline introduction of adjuvant radiotherapy for locally advanced breast cancer (T3/M0 or any T,N2-3/M0), was in 2002 and a large variation existed before and afterwards (Table 3). The control and Northern region hospitals with the highest IP show the best implementation of this recommendation of the guideline while especially before 2008 patients in the other regions were less likely to receive adjuvant radiotherapy.

### Adjuvant chemotherapy for early stage breast cancer

Patients diagnosed in hospitals in the Rotterdam region and Northern Netherlands with a low IP received adjuvant chemotherapy more often for early stage breast cancer than patients in the control hospitals (Table 3). Guideline follow-up in the later time-periods is high and differences between the different hospital categories are small.

### Neo adjuvant chemotherapy for T4/M0 breast cancer

Neo-adjuvant chemotherapy for T4/M0 cancer is administered to approximately half of the patients in the latest time period (Table 3). Because this concerns high stage disease, patient preferences may play an important role in this variation. Both hospital categories in the Rotterdam region as well as the Northern low IP hospitals perform better compared to the control group, with the highest chance of receiving neo adjuvant chemotherapy in the Rotterdam hospitals with high IP (OR 2.67, 95% CI 1.74-4.07, Table 3).

## Discussion

The results of our study show variation in the multidisciplinary treatment of breast cancer patients in the Netherlands. No relationship was evident between variation in multidisciplinary treatment for breast cancer patients and participating in the external peer review programme for multidisciplinary cancer care. In the Northern Netherlands, only the completeness of breast conserving therapy (stadium I-IIIA) was better in patients diagnosed in hospitals with a higher IP compared to the control group. Patients from hospitals with the lowest IP more often received adjuvant chemotherapy for early stage breast cancer, neo-adjuvant chemotherapy for T4 breast cancer and complete breast conserving therapy. In the Rotterdam region, patients diagnosed in hospitals with the highest IP were more likely to receive neo-adjuvant chemotherapy for T4 breast cancer and adjuvant chemotherapy for early stage breast cancer. The latter results also account for patients from hospitals with a low IP from the Rotterdam region when compared to the control group. Differences between the regions imply that there are regional factors that are responsible for the variation.

Before 2002, there was regional variation in guidelines. Table 3 shows that variation decreased in the periods from 2002–2007 and 2008–2010 but no early adopter effect was seen in patients from hospitals with a higher IP. A previous study by van Steenbergen et al. on early stage breast cancer also showed decreased variation after the introduction of national evidence-based guidelines in 2002 but variation still persisted. Differences could be partly explained by hospital characteristics but also by loco-regional practices. Adjuvant systemic therapy was found to be mainly influenced by patient and tumour characteristics [17]. Another study on early stage breast cancer confirms the important role of the national evidence-based guidelines and identified age as the most important factor in the decision whether a patient receives systemic therapy. They also found the presence of early and late-adopters amongst hospitals but could not determine the role of physicians or hospital characteristics [18]. The programmes in the Northern Netherlands and Rotterdam region were similar in origin. During the second cycle in the Rotterdam region, the focus shifted from the evaluation of basic organisational topics to implementing plan-do-check-act cycles and the measurement of quality within hospitals. This shift also occurred in the Northern region but the basic organisational topics remained part of the programme.

The main weakness of our study was that we had to use a black box approach concerning the supposed mechanism through which external peer review on hospital level exerts its influence on tumour service levels. Moreover we did not have the possibility of correcting possible confounding factors such as comorbidity and patient preference. The gradual spread of the programme over the country gave us the possibility to use a control group, creating a quasi-experimental situation. Hospitals in the control group are likely to have introduced changes in their organisation too, but we are not aware of similar programmes that have been used. Hospitals from the high IP and low IP groups may have had different starting points concerning organisational quality, unfortunately we did not have a baseline measurement of organisational quality. Therefore, we can not answer the question if hospitals that already had a good multidisciplinary organisation also performed well on implementing the recommendations from the programme.

Research in this field is challenging. Besides the ‘quasi-experimental’ situation (due to the gradual introduction of the programme) our study had multiple characteristics that helped us to evaluate the impact of external peer review. In the intervention regions all hospitals participated in the programme (even though they did not all give permission to use their data in this study). Because of this, there was no programme participation bias. We did not rank the importance of recommendations to assess the programme impact instead of the impact of single recommendations. We were able to analyse results on a ‘patient level’ because of the reliable and complete data, including information on treatment, over a long period of time provided by the Netherlands Cancer Registry.

## Conclusion

Our study showed regional differences and did not reveal benefits in the multidisciplinary treatment of breast cancer patients being treated in hospitals participating in the programme nor did the extent in which the hospitals implemented the recommendations seem to matter. Organisation focussed quality improvement programmes are generally not designed to directly improve clinical care and in methodological terms this can still be considered as a “black box intervention”. Improving the organisation of care seems a justified goal, but it may be questioned whether the effort put into it is justified if no clinical benefits can be shown. If the objective is that external quality assessment programmes should have a measurable effect on clinical outcomes, the programmes should change their approach. A better focus on the actual delivery of clinical care and incorporating reliable outcome data (from cancer registries) can bridge the gap between quality improvement and patient outcomes. Variation in treatment, as shown in our study can be used as a starting point for quality improvement programmes for hospitals to work on their organisation and delivery of care.

## Electronic supplementary material

Additional file 1: **The external peer review programme for multidisciplinary cancer care in the Netherlands.** (DOCX 40 KB)

## Abbreviations

NCR: Netherlands Cancer Registry
IP: Implementation Proportion
BCT: Breast Conserving Therapy/Treatment
BCS: Breast Conserving Surgery
ALND: Axillary Lymph Node Dissection
SNB: Sentinel Node Biopsy
DCIS: Ductal Carcinoma In Situ.
