# Supplementary material for: Regional variation in breast cancer treatment in the Netherlands and the role of external peer review: a cohort study comprising 63,516 women
Source: BMC Cancer. 2014 Aug 16;14:596. doi: 10.1186/1471-2407-14-596 (PMC4147167; doi:10.1186/1471-2407-14-596)
Supplement: Supplementary file 1 — Additional file 1: The external peer review programme for multidisciplinary cancer care in the Netherlands. (DOCX 40 KB) [file 12885_2013_4778_MOESM1_ESM.docx]

**The external peer review programme for multidisciplinary cancer care in the Netherlands.**

Organisation of cancer care requires agreements between physicians, nursing staff, patients and all others involved. The external peer review programme for multidisciplinary cancer care covers the aspects of care which can be agreed upon to guarantee a high quality of care. This does not concern the actual clinical care but prerequisites that apply to the cancer care organisation. The programme relies on a ‘quality framework’ which describes requirements of three main aspects of care:

-	Policy and organisation: organisational aspects of the hospital itself: internal regulations, means and materials, external cooperation.

-	Management of processes: matters concerning the process that a patient undergoes. For example: shared decision making and exchange of information between providers, referral policies, time till treatment, informational and educational materials.

-	Quality control system: the evaluation and actualisation of the quality control system and measures for improvement

Concerning clinical care: the quality framework describes the requirement of up-to-date guidelines for the treatment of cancer patients. During the external peer review there is no check on an individual patient level if patients received the appropriate treatment. However, cancer registry data is used to evaluate e.g. the use of adjuvant treatments on a hospital level (compared to national averages).
